# Individual digital literacy, area-level digital exclusion, and inequalities in exercise participation among middle-aged and older adults: evidence from China

**DOI:** 10.3389/fpsyg.2026.1907697

**Published:** 2026-10-05

**Authors:** Xiaofan Hao, Meiling Duan, Chenming Zhu, Chuanwen Yu

**Affiliations:** 1 Faculty of Arts and Sports, Huanghe University of Science and Technology, Zhengzhou, Henan, China; 2 Zhengzhou Preschool Education College, Zhengzhou, Henan, China; 3 Dongying Vocational College, Dongying, Shandong, China; 4 School of Sports and Health, Heze University, Heze, Shandong, China

**Keywords:** digital exclusion, digital literacy, exercise participation, health equity, multilevel analysis

## Abstract

**Background:**

Although digital exclusion has been widely discussed in relation to healthcare access and digital service use, less is known about its implications for exercise participation among middle-aged and older adults. This study examined how individual digital literacy and area-level digital exclusion jointly shape exercise participation in China.

**Methods:**

Data were drawn from three waves of the China Health and Retirement Longitudinal Study (CHARLS; 2013, 2015, and 2018) and linked to city-year indicators from the China Statistical Yearbook. The analytic sample included 13,085 person-wave observations from 9,201 unique respondents across 111 cities. Exercise participation was measured as a binary indicator of engagement in exercise-oriented physical activity. Individual digital literacy was assessed using a composite index of digital access, internet use, device resources, and digital behaviors. Area-level digital exclusion was measured using a reverse-coded city-year indicator of contextual digital exclusion. Mixed-effects logistic regression models were used to estimate the associations of individual digital literacy and area-level digital exclusion with exercise participation and to test their cross-level interaction.

**Results:**

37.7% of observations reported exercise participation, increasing from 27.5% in 2013 to 40.3% in 2018. In fully adjusted models, higher individual digital literacy was associated with greater odds of exercise participation (OR = 1.11, 95% CI: 1.022–1.208, *p* < 0.05). Area-level digital exclusion was negatively associated with exercise participation; although the association was attenuated after adjustment for demographic and socioeconomic characteristics, it remained statistically significant in the fully adjusted model (OR = 0.94, 95% CI: 0.869–0.991, *p* < 0.05). The interaction between individual digital literacy and area-level digital exclusion was negative and significant (OR = 0.96, 95% CI: 0.929–0.984, *p* < 0.01), indicating that the positive association between digital literacy and exercise participation was weaker in more digitally excluded cities.

**Conclusion:**

Individual digital literacy was positively associated with exercise participation among middle-aged and older adults in China, whereas area-level digital exclusion weakened this beneficial association. Policies promoting healthy aging should address both older adults’ digital literacy and the broader digital exclusion in which they live.

## Introduction

Population aging is accelerating worldwide, making the promotion of healthy aging a central public health priority. Regular exercise is one of the most important behavioral foundations of healthy aging, as it helps prevent chronic diseases, maintain physical functioning, and improve psychological well-being among middle-aged and older adults (Hu et al., 2024; Craike et al., 2018). However, exercise participation remains unequally distributed. Similar to other health-related behaviors, such as healthy eating, preventive care, health information seeking, and chronic disease self-management, exercise participation is shaped not only by individual motivation and health awareness but also by access to information, socioeconomic resources, supportive environments, and institutional opportunities (Rabbani et al., 2025; Kaihlanen et al., 2022). As societies become increasingly digitalized, these determinants of health behavior are being reshaped by digital technologies. Digital literacy has therefore become an increasingly important resource for health promotion (Yu et al., 2018). Digital technologies, including online health information platforms, mobile applications, wearable devices, social media, and telehealth services, provide new channels through which individuals can obtain health knowledge, receive behavioral guidance, monitor physical activity, and access social support (Xue et al., 2026; Solis-Navarro et al., 2022). For middle-aged and older adults, these technologies may create new opportunities to engage in exercise, manage chronic conditions, and participate in health-promoting activities (Pimenta et al., 2023). At the same time, aging is often accompanied by declining physical function, reduced social networks, and unequal access to technological resources. Many older adults also face barriers in adopting and using digital devices, including limited digital skills, low confidence, affordability constraints, and concerns about the complexity or relevance of digital technologies (Li R. et al., 2025). Thus, the intersections between aging, exercise participation, and digital device usage are increasingly important for understanding inequalities in healthy aging. Digital exclusion has emerged as a major dimension of health inequality. It is now widely understood as a multidimensional phenomenon that extends beyond physical access to digital devices or the internet to include inequalities in digital skills, patterns of meaningful use, and the ability to obtain benefits from digital engagement (Park, 2012; Richardson et al., 2022). In health research, digital exclusion has been associated with disparities in healthcare access, health information seeking, telehealth use, and preventive health behaviors (Mee et al., 2025). Yet relatively less attention has been paid to exercise participation as a behavioral pathway through which digital exclusion may contribute to health inequalities in later life. This omission is important because exercise is a core component of healthy aging and is increasingly embedded in digitally mediated environments, including online exercise information, app-based activity tracking, virtual exercise communities, and platform-based health promotion programmes (Ren and Zhu, 2024).

### Individual digital literacy and exercise participation

At the individual level, digital literacy refers to a person’s ability to access, understand, evaluate, and use digital technologies and resources effectively in everyday life (McDougall et al., 2018). In the context of health promotion, digital literacy may shape whether individuals are able to search for exercise-related information, follow exercise guidance, use digital tools for self-management, and maintain contact with digitally mediated health or exercise resources (Smith et al., 2025). Individuals with higher digital literacy may be better able to obtain reliable exercise information, use mobile applications or wearable devices to monitor physical activity, participate in online exercise communities, and receive personalized guidance through digital health platforms (Li et al., 2026). These advantages may reduce informational barriers, strengthen awareness of exercise, increase motivation, and enhance the feasibility of sustained participation. By contrast, individuals with lower digital literacy may be less able to identify, access, evaluate, and use digital resources related to exercise. They may have difficulty distinguishing reliable health information from misleading content, using digital tools for activity monitoring, or engaging with online health communities. As a result, low digital literacy may reinforce existing inequalities in exercise participation, particularly among middle-aged and older adults who already face socioeconomic disadvantage, poor health, mobility limitations, retirement-related social changes, or limited access to offline exercise support (Wei and Jia, 2025). From this perspective, individual digital literacy can be understood as a personal resource that helps translate digital opportunities into health-promoting behavior. Accordingly, higher individual digital literacy is expected to be associated with a greater likelihood of exercise participation among middle-aged and older adults.

### Area-level digital exclusion and exercise participation

Although individual digital literacy is important, exercise participation also depends on broader contextual conditions. Digital exclusion is not merely an individual-level issue; it is also geographically structured through disparities in digital infrastructure, internet connectivity, technological development, and the availability of digital services across regions (Djatmiko et al., 2025). These area-level differences create unequal digital exclusion that may influence residents’ opportunities to access health information, use digital health services, and benefit from digitally supported exercise resources. In areas where digital exclusion is not a significant issue, residents may have easier access to online health information, digital public services, mobile health applications and community-based digital health programmes (Kaihlanen et al., 2022; Tártaro et al., 2026). Such environments may support exercise participation by reducing the cost of obtaining health information, expanding access to behavioral guidance, strengthening social connections, and facilitating the use of digital tools for health management (Zhou et al., 2025). Conversely, in areas characterized by higher levels of digital exclusion, residents may face limited infrastructure, weaker service provision, and fewer opportunities to obtain and use digital resources for exercise promotion (Yang et al., 2026). These contextual constraints may influence exercise participation independently of individual digital literacy. For middle-aged and older adults, who may already experience age-related barriers to technology use, digital exclusion may further reduce opportunities for exercise participation and widen behavioral health inequalities.

### Cross-level interaction between individual digital literacy and area-level digital exclusion

A multilevel perspective further suggests that the association between individual digital literacy and exercise participation may depend on the broader digital exclusion in which individuals are embedded (Chen and Xiao, 2024). The same level of digital literacy may not generate the same behavioral outcome across different regional contexts. In more supportive digital environments, individuals with higher digital literacy may be better able to convert their skills and resources into actual exercise participation because digital information, services, and supportive tools are more readily available (Li H. et al., 2025). In such contexts, individual digital literacy can be more effectively translated into health-promoting behavior. In contrast, in areas characterized by greater digital exclusion, even individuals with relatively strong digital literacy may face limited opportunities to apply their skills to exercise-related activities. Poor connectivity, limited availability of digital health services, and underdeveloped technological conditions may constrain the practical value of individual digital literacy (Mohammadyari and Singh, 2015). This implies that contextual digital exclusion may weaken the positive role of individual digital literacy. Digital exclusion should therefore be understood not only as the sum of individual and contextual disadvantages but also as a cross-level process in which area-level digital conditions shape the extent to which individual digital literacy can be translated into exercise participation (Sharma et al., 2016). Understanding this cross-level interaction is essential for explaining how digital exclusion operates as a multilevel mechanism shaping inequalities in health behavior. Although existing studies have increasingly recognized the importance of digital health equity, most have focused either on individual digital factors or on contextual digital inequalities (Zhou et al., 2026). Few studies have examined how individual digital literacy and area-level digital exclusion jointly shape health-related behaviors among aging populations. As a result, it remains unclear whether disadvantaged digital exclusion weaken, strengthen, or otherwise modify the relationship between individual digital literacy and exercise participation. This gap limits our understanding of how digital exclusion contributes to unequal health behaviors in later life and constrains the development of targeted interventions that address both individual capabilities and contextual barriers.

### Conceptual framework and research hypotheses

Using data from CHARLS and city-level indicators from the China Statistical Yearbook, this study applies a multilevel framework to examine how individual digital literacy and area-level digital exclusion are associated with exercise participation among middle-aged and older adults in China. Individual digital literacy is operationalized as a proxy-based construct derived from internet access, frequency of internet use, device-related resources, and selected digital behaviors available in CHARLS. Although CHARLS does not include a standardized digital literacy scale, this approach provides a defensible way to capture variation in digital engagement and capacity. This study has three aims. First, it examines whether individual digital literacy is positively associated with exercise participation. Second, it investigates whether area-level digital exclusion is negatively associated with exercise participation. Third, it assesses whether area-level digital exclusion weakens the association between individual digital literacy and exercise participation. Accordingly, we propose the following hypotheses: H1: individual digital literacy is positively associated with exercise participation; H2: area-level digital exclusion is negatively associated with exercise participation; and H3: area-level digital exclusion negatively moderates the association between individual digital literacy and exercise participation, such that the positive association is weaker in areas with higher levels of digital exclusion.

By integrating individual-level digital capacity with area-level digital conditions, this study makes three contributions. First, it extends research on digital literacy and health behaviors by focusing on exercise participation, an important but underexplored behavioral outcome in studies of digital inequality and healthy aging. Second, it moves beyond an individual-centered perspective by highlighting the role of the digital exclusion. Third, it tests a cross-level interaction between individual digital literacy and area-level digital exclusion, thereby providing evidence on how digital resources may generate unequal behavioral returns across local contexts. Figure 1 presents the conceptual framework guiding the empirical analysis.

**FIGURE 1:**
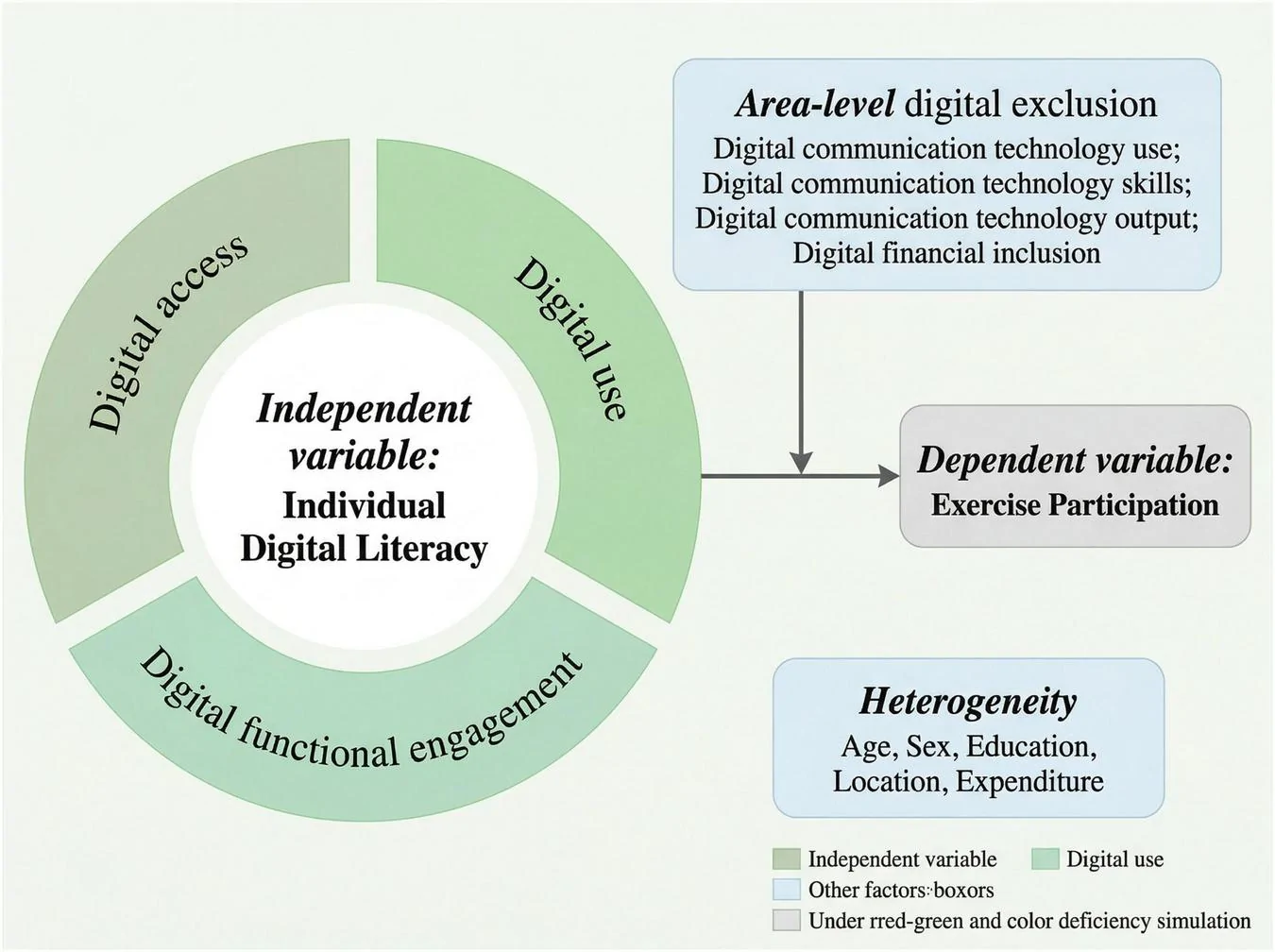
Theoretical analysis diagram.

## Materials and methods

### Data source and study sample

The individual-level data were derived from the CHARLS, a nationally representative longitudinal survey of middle-aged and older adults in China. CHARLS collects detailed information on demographic characteristics, socioeconomic conditions, health status, family resources, and community context. This study used data from three waves of CHARLS conducted in 2013, 2015, and 2018, and linked these individual-level data to external city-year indicators derived from the China Statistical Yearbook. It should be noted that CHARLS was not conducted as an online survey. The study sample was restricted to respondents aged 45 years and older, consistent with the sampling design of CHARLS and with the focus of this study on middle-aged and older adults. This age range is appropriate because midlife and later life are critical periods for chronic disease prevention, functional maintenance, exercise behavior, and adaptation to digital technologies. Including adults aged 45 and older therefore allows this study to examine exercise participation across the transition from midlife to later life. For this study, the original wave-specific CHARLS files were used for all three waves to ensure consistency in variable construction and harmonization across survey years. Respondents were linked across waves using individual identifiers, while household and community identifiers were retained to facilitate household- and area-level matching. The analytic sample included respondents aged 45 years and older who had valid information on the main outcome, individual digital literacy, and key covariates in at least one survey wave. Observations with missing information on the dependent variable were excluded from the corresponding wave-specific analyses. After data restructuring, the final dataset was organized in long format, with repeated observations nested within individuals and individuals nested within cities. After data cleaning and the exclusion of observations with missing values on the main variables, the final analytic sample included 13,085 person-wave observations from 9,201 unique respondents across 111 cities in the 2013, 2015, and 2018 waves. All respondents provided informed consent during data collection, and no personally identifiable information was included in this analysis.

### Measures

#### Exercise participation

The main outcome was exercise participation, defined to capture health-promoting physical activity rather than labor-related activity. This distinction is important because CHARLS records vigorous, moderate, and light activities arising from occupational, domestic, recreational, and exercise-related purposes. To avoid conflating exercise with physically demanding work, this study focused on activities explicitly reported as being undertaken for exercise. In each survey wave, respondents were asked whether they had engaged in vigorous, moderate, or light physical activity lasting at least 10 continuous minutes, how many days per week they engaged in such activity, how long the activity usually lasted, and the main purpose of the activity. Based on these items, a binary indicator of exercise participation was constructed and coded as 1 if the respondent reported any vigorous, moderate, or light activity for exercise purposes, and 0 otherwise.

#### Individual digital literacy

Because CHARLS does not include a validated standardized scale of digital literacy, individual digital literacy was operationalized using a proxy-based composite measure derived from the digital indicators available consistently across survey waves. The measure was intended to capture respondents’ relative digital access, use, and functional engagement rather than digital literacy skills in the strict psychometric sense.

The construction followed a multidimensional logic. Digital access was represented by internet use and household ownership of digital devices, including mobile phones and computers. Digital-use intensity reflected the frequency with which respondents used the internet. Where available, additional digital behaviors were used to characterize functional engagement with digital technologies, including activities such as obtaining information, watching online content, using financial or payment services, and engaging with social media.

For the main longitudinal analysis, we constructed a parsimonious core index using indicators that were consistently available in the 2013, 2015, and 2018 waves, including internet use, frequency of internet use, household mobile-phone ownership, and household computer ownership. Component indicators were coded so that higher values represented greater digital engagement and were standardized within each survey wave before aggregation. Thus, standardization was performed within wave rather than across the pooled analytic sample. This approach was used because the availability and prevalence of digital technologies changed substantially over the study period; within-wave standardization therefore captures respondents’ relative digital position within the technological environment prevailing at each survey wave.

Importantly, this composite should be interpreted as a proxy for individual digital capacity rather than a direct psychometric assessment of digital literacy. Because some components capture access to digital devices and resources, the index may also reflect socioeconomic circumstances, household technological resources, and other characteristics associated with digital engagement. Multivariable models therefore adjusted for major demographic and socioeconomic covariates, and sensitivity analyses used alternative digital-literacy specifications, including an extended index incorporating richer digital-behavior indicators available in the later survey wave.

#### Area-level digital exclusion

Area-level digital exclusion was measured by matching an external city-year indicator to respondents’ geographic location and survey year. This measure was intended to capture contextual disparities in digital infrastructure, digital connectivity, and the broader local digital environment that may shape opportunities for translating individual digital literacy into exercise participation. Following previous Chinese research on regional digital exclusion, the city-level measure was constructed from four dimensions of digital development, including digital communication technology use, digital communication technology skills, digital communication technology output, and digital financial inclusion. Based on data availability, these dimensions were operationalized using the number of internet users per 100 persons, the number of higher education students enrolled, per capita total telecommunications business volume, and the Digital Financial Inclusion Index, respectively. Because sufficient data were not available for the original indicator of higher education penetration, the number of students enrolled in higher education institutions was used as a proxy. To capture inter-city disparity in digital development, an improved location quotient approach was used to compare each city’s digital technology development level with the national average. The specific calculation formula is as follows:


$$D\,i\,g\,D\,Q_{i}=\frac{D\,i\,g\,D_{i}}{D\,i\,g\,D}=\frac{\sum_{j=1}^{m}q_{i\,j}}{\sum_{j}^{m}Q_{j}}$$

*q*_*ij*_ represents the j-th secondary indicator value for the i-th city, and *Q_j_* represents the j-th secondary indicator value for China. Where i = 1,…, n; j = 1,…, m. n represents the number of cities, and m represents 4 secondary indicators. *DigDQ_i_* represents the location entropy of the digital technology development index for the i-th city.

The resulting index (*DigDQ_i_*) reflects the relative digital development position of city i . After calculating the location quotient, the resulting values were standardized before aggregation. Because the original location quotient primarily indicates relative digital development advantage, the measure was reverse-coded in the main analysis so that higher values represented greater area-level digital exclusion, that is, greater contextual digital exclusion. The city-year digital exclusion measure was then merged with the CHARLS data using respondents’ city and survey year. After merging, each respondent-wave observation was assigned the value corresponding to the city-year context in which the respondent was located.

#### Control variable

A set of demographic and socioeconomic covariates was included to reduce confounding. These included age, sex, educational attainment, marital status, rural-urban residence, and expenditure. Sex and education were treated as relatively stable background characteristics, whereas age, marital status, rural-urban residence, and expenditure were treated as time-varying covariates measured separately in each survey wave.

### Statistical analysis

The analysis proceeded in four steps. First, descriptive statistics were used to summarize the distribution of exercise participation, individual digital literacy, area-level digital exclusion, and other covariates across survey waves. Second, Second, bivariate associations between the main explanatory variables and exercise participation were examined. For individual digital literacy and area-level digital exclusion, respondents were divided into low, medium, and high groups. The number and percentage of exercise participants were reported for each group. Group differences in exercise participation were first assessed using chi-square tests. In addition, unadjusted generalized estimating equation (GEE) logistic regression models were used to estimate odds ratios (ORs), 95% confidence intervals (CIs), and *p*-values for the association between each explanatory variable and exercise participation, with the lowest group used as the reference category. The GEE approach was used to account for the correlation among repeated observations from the same individuals across survey waves. Third, the main hypotheses were tested using mixed-effects logistic regression models. Because repeated observations were nested within individuals and individuals were nested within cities, a multilevel modeling strategy was adopted to account for both within-person correlation over time and contextual clustering at the city level. Since the main dependent variable, exercise participation, was binary, mixed-effects logistic regression was used as the primary analytical approach. The general specification was:


$$l\,o\,g\,i\,t\,\left[P\,\left(E\,x\,e\,r\,c\,i\,s\,e\,P\,a\,r\,t_{t\,i\,j}=1\right)\right]$$

$$=\beta_{0}+\beta_{1}\,D\,i\,g\,i\,t\,a\,l\,L\,i\,t_{t\,i\,j}+\beta_{2}\,D\,i\,g\,i\,t\,a\,l\,E\,x\,c\,l_{t\,j}$$

$$+\beta_{3}\,\left({\mathrm{DigitalLit}}_{\mathrm{tij}}\times {\mathrm{DigitalExcl}}_{\mathrm{tj}}\right)+\beta_{4}\,X_{\mathrm{tij}}+\lambda_{t}+\mu_{i}+v_{j}+\varepsilon_{\mathrm{tij}}$$

where *t* denotes survey wave, *i* denotes individual, and *j* denotes city. *ExercisePart*_*tij*_ represents the binary exercise participation outcome; *DigitalLit*_*tij*_ is the individual digital literacy index; *DigitalExcl*_*tj*_ is the area-level digital exclusion indicator; and *X*_*tij*_ is a vector of individual-level covariates. λ_*t*_ represents survey-wave fixed effects, μ_*i*_ captures unobserved individual-level heterogeneity, and *v*_*j*_ captures city-level random effects. The coefficient of greatest substantive interest was the interaction term between individual digital literacy and area-level digital exclusion. A negative interaction coefficient would indicate that greater area-level digital exclusion weakens the positive association between individual digital literacy and exercise participation. By contrast, a positive interaction coefficient would suggest that individual digital literacy becomes especially important under conditions of greater contextual digital exclusion, implying a compensatory effect of individual digital resources.

### Sensitivity analyses

To assess the robustness of the main findings, several sensitivity analyses were conducted. First, the main models were re-estimated using regular exercise rather than any exercise participation as the dependent variable, in order to examine whether the results were sensitive to a stricter definition of exercise engagement. Second, alternative specifications of individual digital literacy were tested, including simpler additive indices and extended indices incorporating richer digital behavior items available in later survey waves. Third, robustness checks were performed using a supplementary indicator of low activity risk based on approximate weekly exercise time. Fourth, because CHARLS provides wave-specific cross-sectional weights rather than a unified panel weight, the main mixed-effects logistic regression models were estimated without imposing a single cross-sectional weighting structure, while weighted descriptive statistics and wave-specific comparisons were retained to improve representativeness. The data supporting the findings of this study are provided in Supplementary Data 1.

## Results

### Descriptive characteristics of the analytic sample

The statistics presented in Table 1 are weighted descriptive estimates and weighted wave-specific comparisons based on the CHARLS cross-sectional individual weights. By contrast, the main mixed-effects logistic regression models were estimated without imposing a single weighting structure, because CHARLS provides wave-specific cross-sectional weights rather than a unified longitudinal panel weight. This approach allowed us to retain representativeness in the descriptive analyses while avoiding potential misspecification in the longitudinal multilevel models. After excluding observations with missing values on key variables, 9,034 respondents with valid CHARLS cross-sectional weights were included, of which 1,356 were from 2013, 1,745 from 2015, and 5,933 from 2018. Overall, 37.7% of observations reported exercise participation, and this proportion increased across survey waves, from 27.5% in 2013 to 36.3% in 2015 and 40.3% in 2018 (*p* < 0.001). The mean digital core score was 0.6 (SD 0.3) overall and differed significantly by wave, with lower values in 2013 and 2015 than in 2018 (*p* < 0.001). City-level digital exclusion also varied significantly across waves, with a mean of −0.2 (SD 1.1) overall (*p* < 0.001). Respondents had a mean age of 58.7 years (SD 9.0), and age increased modestly over time (*p* < 0.001). Annual household expenditure also differed across waves (*p* = 0.047). The sample was broadly balanced by sex (49.2% female), while educational attainment, marital status, and exercise participation showed significant variation across survey waves (all *p* < 0.001). Most respondents lived in villages (67.8%) and were married (89.7%). Residence was the only characteristic that did not differ significantly across waves at the conventional 5% level (*p* = 0.051). Figure 2 further illustrates the distribution of the sample population and the digital exclusion across Chinese cities in 2013, 2015, and 2018, showing clear spatial and temporal heterogeneity in contextual digital conditions.

**TABLE 1:** Weighted descriptive characteristics of the analytic sample by survey wave.

Variable	Level	Overall	2013	2015	2018	*P*
*n*		9034	1356	1745	5933	
Digital core [mean (SD)]		0.6 (0.3)	1.0 (0.0)	1.0 (0.1)	1.4 (0.2)	<0.001
Exercise participation (%)	No	6032 (62.3)	1036 (72.5)	1192 (63.7)	3804 (59.7)	<0.001
	Yes	3002 (37.7)	320 (27.5)	553 (36.3)	2129 (40.3)	<0.001
Age [mean (SD)]		58.7 (9.0)	57.9 (9.0)	58.0 (8.8)	59.0 (9.1)	<0.001
Annual household expenditure [mean (SD)]		12425.1 (14563.7)	13368.6 (15975.7)	13065.9 (13790.4)	12052.3 (14430.4)	0.047
Sex (%)	Female	4514 (49.2)	679 (50.9)	816 (45.9)	3019 (49.7)	0.044
	Male	4520 (50.8)	677 (49.1)	929 (54.1)	2914 (50.3)	0.044
Education (%)	Primary school or below	5400 (55.5)	745 (51.2)	978 (50.9)	3677 (57.6)	<0.001
	High school	3157 (36.8)	530 (41.8)	655 (39.1)	1972 (35.1)	<0.001
	College or above	477 (7.7)	81 (7.1)	112 (9.9)	284 (7.3)	<0.001
Location (%)	Village	6930 (67.8)	996 (66.4)	1295 (64.9)	4639 (68.9)	0.051
	City/town	2104 (32.2)	360 (33.6)	450 (35.1)	1294 (31.1)	0.051
Marital (%)	Unmarried	871 (10.3)	187 (14.6)	199 (11.3)	485 (9.1)	<0.001
	Married	8163 (89.7)	1169 (85.4)	1546 (88.7)	5448 (90.9)	<0.001
Digital exclusion city [mean (SD)]		−0.2 (1.1)	0.2 (1.0)	−0.3 (1.3)	−0.2 (1.1)	<0.001

Descriptive statistics are weighted using CHARLS wave-specific cross-sectional individual weights (INDV_weight_ad2) and are based on 9,034 respondents with valid weights. Continuous variables are shown as weighted means (SD), and categorical variables as unweighted *n* (weighted %). *P*-values are from weighted comparisons across survey waves. The main regression analyses used 13,085 person-wave observations from 9,201 unique respondents across 111 cities and were estimated without cross-sectional weights because CHARLS does not provide a unified longitudinal panel weight.

**FIGURE 2:**
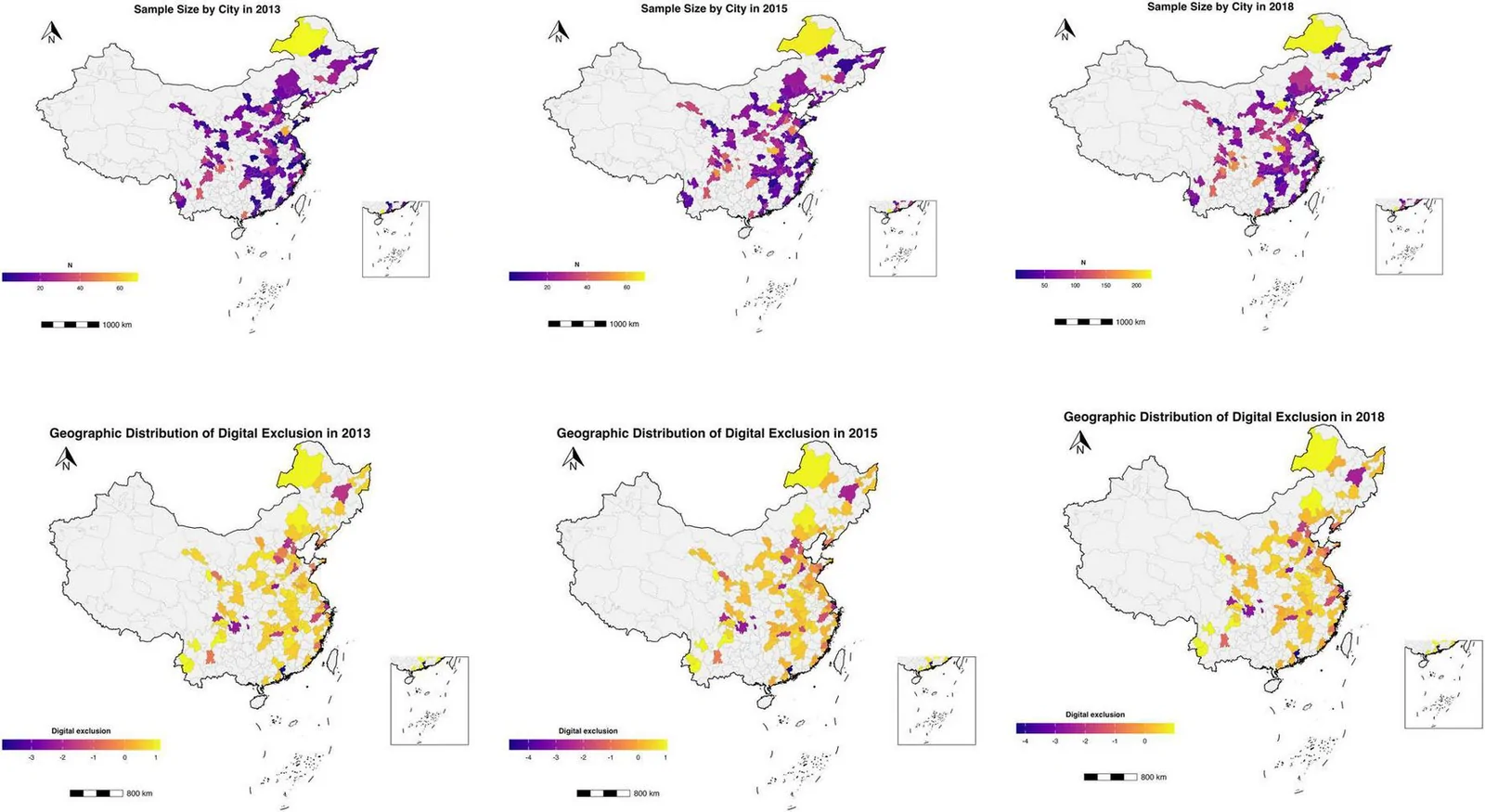
Distribution of the sample population and the digital divide in 2013, 2015 and 2018.

### Bivariate associations between digital exclusion and exercise participation

Table 2 presents the bivariate associations between digital exclusion and exercise participation. When respondents were grouped by levels of individual digital literacy, exercise participation differed significantly across groups (χ^2^ *p* < 0.001). Among respondents with low individual digital literacy, 34.1% participated in exercise; among those with medium digital literacy, the corresponding proportion was 35.7%; and among those with high digital literacy, the proportion was 30.2%. Using the low digital literacy group as the reference category, the unadjusted GEE results showed that the odds of exercise participation were not significantly different for the medium digital literacy group (OR = 1.06, 95% CI: 0.97–1.15, *p* = 0.217), whereas the high digital literacy group showed significantly lower odds of exercise participation (OR = 0.78, 95% CI: 0.71–0.85, *p* < 0.001). By contrast, the bivariate pattern for area-level digital exclusion was more consistent. Exercise participation declined as area-level digital exclusion increased (χ^2^ *p* < 0.001). Specifically, 36.3% of respondents in the low digital exclusion group participated in exercise, compared with 33.8% in the medium digital exclusion group and 29.9% in the high digital exclusion group. Relative to the low exclusion group, the unadjusted GEE estimates showed that respondents in cities with medium levels of digital exclusion had lower odds of exercise participation (OR = 0.91, 95% CI: 0.83–1.00, *p* = 0.043), and those in cities with high levels of digital exclusion had substantially lower odds (OR = 0.74, 95% CI: 0.67–0.81, *p* < 0.001). Importantly, the bivariate association between the proxy-based digital literacy index and exercise participation did not follow a monotonic gradient. Although exercise participation was slightly higher in the medium group than in the low group, respondents in the high group showed lower participation in the unadjusted comparison. This pattern should not be interpreted as evidence that greater digital literacy reduces exercise participation. Rather, because these estimates do not adjust for differences in age, survey wave, education, residence, socioeconomic resources, health-related characteristics, or other compositional factors, the grouped comparison may reflect substantial confounding. In addition, the composite index captures not only digital use but also access to household technological resources, which may correlate with characteristics that independently influence exercise behavior. The Table 2 results are therefore treated as descriptive rather than as evidence of a dose–response association. Multivariable mixed-effects models were subsequently used to estimate the conditional association between the continuous digital-literacy proxy and exercise participation after adjustment for observed demographic and socioeconomic characteristics.

**TABLE 2:** Bivariate associations of individual digital literacy and area-level digital exclusion with exercise participation among middle-aged and older adults.

Variable	Group	*N*	Exercise participants	Exercise participatio*n* (%)	χ^2^ *P*-value	Unadjusted GEE OR (95% CI)	GEE *p*
Individual digital literacy	Low	4362	1486	34.1	<0.001	Ref	
Individual digital literacy	Medium	4362	1557	35.7		1.06 (0.97, 1.15)	0.217
Individual digital literacy	High	4361	1319	30.2		0.78 (0.71, 0.85)	<0.001
Area-level digital exclusion	Low	4362	1583	36.3	<0.001	Ref	
Area-level digital exclusion	Medium	4362	1476	33.8		0.91 (0.83, 1.00)	0.043
Area-level digital exclusion	High	4361	1303	29.9		0.74 (0.67, 0.81)	<0.001

Cells report the number and percentage of exercise participants in each group. Group differences in exercise participation were assessed using chi-square tests. Odds ratios (ORs), 95% confidence intervals (CIs), and *p*-values were estimated using unadjusted generalized estimating equation (GEE) logistic regression models, with the lowest group as the reference category. GEE models accounted for repeated observations from the same individuals across survey waves.

### Mixed-effects logistic regression results

Table 3 presents the mixed-effects logistic regression results for exercise participation among middle-aged and older adults. Model 1 includes survey-wave indicators only. Model 2 adds individual digital literacy, Model 3 further introduces area-level digital exclusion, Model 4 adjusts for demographic and socioeconomic covariates, and Model 5 additionally includes the interaction term between individual digital literacy and area-level digital exclusion. Individual digital literacy was positively associated with exercise participation. In Model 2, a one-unit increase in the composite individual digital-literacy index was associated with 16% higher odds of exercise participation (OR = 1.16, 95% CI: 1.074–1.250, *p* < 0.001). This positive association remained stable after the inclusion of area-level digital exclusion and other covariates. In the fully adjusted model (Model 5), the odds ratio for individual digital literacy was 1.11 (95% CI: 1.022–1.208, *p* < 0.05). These findings indicate that respondents with higher levels of digital literacy were more likely to participate in exercise, supporting H1.

**TABLE 3:** Mixed-effects logistic regression results for exercise participation.

Variable	Model 1	Model 2	Model 3	Model 4	Model 5
Year: 2015 (vs. 2013)	1.54 (1.331, 1.787)***	1.54 (1.332, 1.787)***	1.45 (1.245, 1.689)***	1.60 (1.367, 1.861)***	1.54 (1.321, 1.800)***
Year: 2018 (vs. 2013)	2.06 (1.818, 2.328)***	2.67 (2.224, 3.202)***	2.51 (2.081, 3.026)***	2.67 (2.197, 3.256)***	2.56 (2.101, 3.115)***
Individual digital literacy		1.16 (1.074, 1.250)***	1.16 (1.072, 1.248)***	1.12 (1.030, 1.218)**	1.11 (1.022, 1.208)*
Area-level digital exclusion			0.84 (0.750, 0.945)**	0.94 (0.870, 0.987)*	0.94 (0.869, 0.991)*
Age				1.03 (1.029, 1.040)***	1.03 (1.028, 1.039)***
Sex				0.85 (0.778, 0.929)***	0.85 (0.781, 0.932)***
Education				1.25 (1.142, 1.360)***	1.24 (1.137, 1.353)***
Location				3.13 (2.779, 3.523)***	3.13 (2.784, 3.528)***
Marital				0.98 (0.844, 1.128)	0.97 (0.841, 1.123)
Expenditure				1.11 (1.050, 1.169)***	1.09 (1.036, 1.153)***
Digital literacy × digital exclusion					0.96 (0.929, 0.984)**
Observations	13085	13085	13085	13085	13085
Individuals	9201	9201	9201	9201	9201
Cities	111	111	111	111	111
Random intercept variance (ID)	0.607	0.603	0.605	0.559	0.544
Random intercept variance (city name)	0.356	0.336	0.305	0.108	0.108
AIC	15948.8	15937.9	15931.9	15257.7	15259.8
BIC	15986.2	15982.8	15984.3	15355.4	15362.5

*p < 0.05, **p < 0.01, ***p < 0.001. Cells report OR (95% CI) and *p*-value.

Area-level digital exclusion was negatively associated with exercise participation in Model 3 (OR = 0.84, 95% CI: 0.750–0.945, *p* < 0.01), suggesting that respondents living in more digitally excluded cities were less likely to participate in exercise. After demographic and socioeconomic covariates were introduced, this association was attenuated but remained statistically significant in Models 4 and 5. In the fully adjusted model, higher area-level digital exclusion was still associated with lower odds of exercise participation (Model 5: OR = 0.94, 95% CI: 0.869–0.991, *p* < 0.05). This pattern indicates that part of the association between contextual digital exclusion and exercise participation can be explained by individual demographic and socioeconomic characteristics, but that area-level digital exclusion retains an independent negative association with exercise participation after adjustment. Accordingly, H2 was supported.

Among the control variables, age was positively associated with exercise participation (Model 5: OR = 1.03, 95% CI: 1.028–1.039, *p* < 0.001). Education also showed a significant positive association (OR = 1.24, 95% CI: 1.137–1.353, *p* < 0.001), as did expenditure (OR = 1.09, 95% CI: 1.036–1.153, *p* < 0.01). In addition, location was strongly associated with exercise participation (OR = 3.13, 95% CI: 2.784–3.528, *p* < 0.001), indicating substantial differences by place of residence. By contrast, sex was negatively associated with exercise participation (OR = 0.85, 95% CI: 0.781–0.932, *p* < 0.001), whereas marital status was not statistically significant. The survey-wave coefficients further suggest a temporal increase in exercise participation. Compared with 2013, respondents in 2015 had significantly higher odds of exercise participation (Model 5: OR = 1.54, 95% CI: 1.321–1.800, *p* < 0.001), and the increase was even greater in 2018 (OR = 2.56, 95% CI: 2.101–3.115, *p* < 0.001). This indicates that exercise participation rose over time during the study period. The random intercept variances suggest that unobserved heterogeneity remained at both the individual and city levels. After covariates were included, the city-level variance declined from 0.356 in Model 1 to 0.108 in Models 4 and 5, while the individual-level variance declined from 0.607 to 0.544. This pattern suggests that part of the between-city variation in exercise participation was explained by observed variables, whereas substantial between-individual heterogeneity remained.

### Cross-level interaction between individual digital literacy and area-level digital exclusion

As shown in Table 3, Model 5 added the interaction between individual digital literacy and area-level digital exclusion. The interaction term was negative and statistically significant (OR = 0.96, 95% CI: 0.929–0.984, *p* < 0.01), indicating a modest attenuation in the association between individual digital literacy and exercise participation as area-level digital exclusion increased.

However, model-fit statistics did not indicate an improvement after inclusion of the interaction term. The AIC increased slightly from 15,257.7 in Model 4 to 15,259.8 in Model 5, while the BIC increased from 15,355.4 to 15,362.5. Thus, although the interaction was statistically significant and consistent with the hypothesized direction, the model including the interaction was not preferred by parsimony-based criteria, suggesting that its incremental contribution to overall model fit was limited.

Moreover, the magnitude of the interaction was small on the multiplicative odds scale. An interaction OR of 0.96 corresponds to approximately a 4% attenuation in the digital-literacy odds ratio for each one-unit increase in area-level digital exclusion. Because interaction effects in logistic models depend on the underlying covariate values and baseline probability of the outcome, practical significance cannot be inferred from the odds ratio alone. Figure 3 therefore presents predicted probabilities to illustrate the interaction on an absolute probability scale. The figure indicates that the positive slope relating digital literacy to exercise participation becomes flatter at higher levels of area-level digital exclusion, although the magnitude of this difference is modest.

**FIGURE 3:**
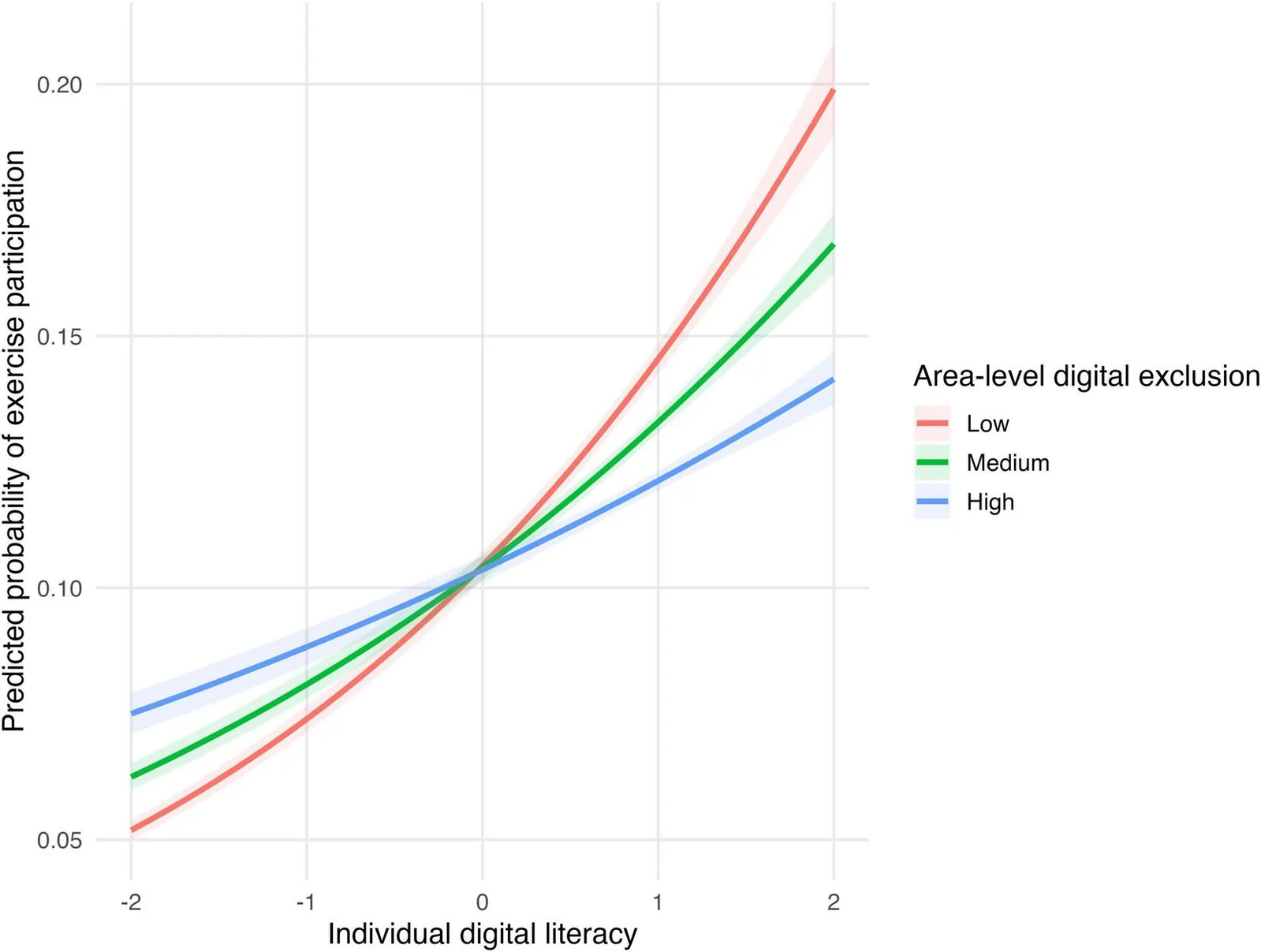
Predicted probabilities of exercise participation by individual digital literacy across levels of area-level digital exclusion. Predicted probabilities were estimated from the mixed-effects logistic regression model. Low, medium, and high area-level digital exclusion correspond to –1 SD, mean, and +1 SD, respectively.

### Heterogeneity analysis

Heterogeneity analyses are presented in Table 4. Formal between-group tests indicated that the clearest evidence of heterogeneity concerned age. In particular, the interaction between individual digital literacy and area-level digital exclusion differed between respondents aged 45–59 years and those aged 60 years and older (p for difference = 0.002), with a stronger negative interaction observed among older adults. By contrast, although subgroup-specific point estimates varied across residence and educational groups, formal tests provided limited evidence that these associations differed statistically between groups. For household economic resources, the association between individual digital literacy and exercise participation appeared to vary across expenditure groups (p for difference = 0.028), whereas evidence of heterogeneity in the area-level digital exclusion effect and the cross-level interaction was weaker. Given the number of subgroup comparisons, these findings should be interpreted as exploratory.

**TABLE 4:** Heterogeneity analyses across sociodemographic subgroups.

Stratifier	Subgroup	Observations	Digital literacy OR (95% CI)	Digital exclusion OR (95% CI)	Digital literacy × digital exclusion OR (95% CI)	P-difference: digital literacy	P-difference: digital exclusion	P-difference: interaction
Age	45–59 years	7,552	1.05 (0.75, 1.48)	0.88 (0.75, 1.03)	1.11 (0.91, 1.34)	0.165	0.010	0.002
	≥60 years	5,533	1.64 (1.07, 2.51)	1.11 (0.97, 1.26)	0.75 (0.63, 0.89)			
Residence	Rural	10,003	1.59 (1.15, 2.19)	0.94 (0.83, 1.06)	0.93 (0.80, 1.07)	0.892	0.071	0.514
	Urban	3,082	1.54 (0.98, 2.40)	1.20 (0.98, 1.47)	0.83 (0.65, 1.06)			
Education	Higher education	4,921	1.36 (0.95, 1.94)	0.96 (0.81, 1.15)	0.96 (0.77, 1.18)	0.214	0.721	0.532
	Low education	8,164	1.98 (1.35, 2.92)	1.00 (0.88, 1.14)	0.88 (0.75, 1.04)			
Household economic resources	High	4,361	1.20 (1.01, 1.43)	0.96 (0.85, 1.09)	0.98 (0.87, 1.10)	0.028	0.064	0.184
	Low	4,362	1.51 (0.94, 2.43)	1.17 (0.97, 1.41)	0.81 (0.62, 1.05)			
	Middle	4,362	1.93 (1.43, 2.60)	0.89 (0.78, 1.02)	0.91 (0.79, 1.05)			

### Robustness tests

Table 5 presents robustness checks using alternative measures of digital literacy and exercise outcomes. In Model 1, where the dependent variable was replaced with regular exercise, individual digital literacy remained positively associated with the outcome (OR = 1.29, 95% CI: 1.00–1.66), whereas area-level digital exclusion was negatively associated with regular exercise (OR = 0.86, 95% CI: 0.75–0.96). In Model 2, which used the extended digital literacy index estimated on the 2018 subsample, the results remained broadly consistent: individual digital literacy was positively associated with exercise-related outcomes (OR = 1.49, 95% CI: 1.10–2.02), while area-level digital exclusion again showed a negative association (OR = 0.97, 95% CI: 0.85–0.98). In Model 3, using ordered exercise level as the dependent variable also produced substantively similar results, with a positive coefficient for individual digital literacy (OR = 1.34, 95% CI: 1.05–1.71) and a negative coefficient for area-level digital exclusion (OR = 0.95, 95% CI: 0.86–0.99). Notably, the interaction between individual digital literacy and area-level digital exclusion remained negative and statistically significant across all three models, with odds ratios ranging from 0.86 to 0.89. Taken together, these results indicate that the main finding regarding the negative moderating role of contextual digital exclusion is robust to alternative operationalizations of both digital literacy and exercise outcomes.

**TABLE 5:** Results of robustness checks.

Variable	Model 1: regular exercise	Model 2: extended digital literacy index (2018 only)	Model 3: ordered exercise level
Individual digital literacy	1.29 (1.00, 1.66)*	1.49 (1.10, 2.02)**	1.34 (1.05, 1.71)**
Area-level digital exclusion	0.86 (0.750, 0.96)**	0.97 (0.85, 0.98)*	0.95 (0.86, 0.99)*
Digital literacy × digital exclusion	0.89 (0.79, 0.99)*	0.86 (0.78, 0.95)*	0.88 (0.81, 0.96)**
Year 2015 (vs. reference year)			1.54 (1.32, 1.79)***
Year 2018 (vs. reference year)			2.38 (1.96, 2.88)***
Observations	13085	8698	13085
Individuals	9201		9201
Cities	111	111	111
Random intercept variance (ID)	0.547		0.590
Random intercept variance (city name)	0.107	0.101	0.095
AIC	14939.7	10696.0	22100.03

Model 1 uses regular exercise as the dependent variable. Model 2 replaces the baseline digital literacy measure with the extended digital literacy index and is estimated on the later-wave subsample with non-missing extended digital literacy items. Model 3 uses ordered exercise level (0 = no exercise, 1 = irregular exercise, 2 = regular exercise, 3 = regular exercise with moderate-to-vigorous intensity) and is estimated using a cumulative link mixed model (clmm). Entries are odds ratios (ORs) with 95% confidence intervals (CIs); estimates in Model 3 are cumulative odds ratios. Random intercepts are included at the individual and/or city level as applicable. Threshold coefficients are omitted for brevity. *p < 0.05, **p < 0.01, ***p < 0.001.

## Discussion

This study examined the associations of individual digital literacy and area-level digital exclusion with exercise participation among middle-aged and older adults in China. Three main findings emerged. First, higher individual digital literacy was associated with a greater likelihood of exercise participation after adjustment for demographic, socioeconomic, temporal, and contextual characteristics. Second, higher area-level digital exclusion was associated with lower exercise participation, although this association was attenuated after adjustment for individual characteristics. Third, area-level digital exclusion modestly weakened the positive association between individual digital literacy and exercise participation.

Exercise participation increased descriptively across the three survey waves. Although survey-wave indicators were included in the regression models to account for temporal differences, the present study was not designed to identify the factors responsible for this temporal pattern. The observed increase may reflect broader social, environmental, behavioral, or policy changes that were not directly measured in this study and should therefore be interpreted as descriptive context rather than evidence of a specific underlying mechanism. More importantly, the positive adjusted association between individual digital literacy and exercise participation is broadly consistent with research suggesting that digital resources may facilitate access to health information, preventive behaviors, self-management, and health services (Wang et al., 2026; Jafar et al., 2024; Tian, 2025). Our findings extend this literature by suggesting that digital capacity may also be relevant to exercise participation, an important component of healthy aging. For middle-aged and older adults, greater digital capacity may facilitate access to exercise-related information, online guidance, digital health tools, and socially mediated health resources, although these potential mechanisms were not directly tested in the present study.

The contrast between the unadjusted and adjusted results deserves particular attention. In the bivariate analysis, exercise participation did not increase monotonically across categories of the digital-literacy proxy, and the high group showed lower exercise participation than the low group. After adjustment for demographic, socioeconomic, temporal, and contextual characteristics, however, the continuous digital-literacy proxy was positively associated with exercise participation. This reversal suggests that the crude group differences were influenced by differences in the composition of the digital-literacy groups rather than reflecting a simple dose–response relationship. Individuals with different levels of digital engagement may also differ in age, education, residence, socioeconomic resources, health status, and patterns of technology adoption, all of which may independently influence exercise participation. The adjusted association should therefore be interpreted as conditional on the covariates included in the models rather than as evidence of a direct causal effect of digital literacy.

Area-level digital exclusion was also associated with exercise participation, although its association was reduced after individual characteristics were taken into account. This attenuation suggests that contextual digital disadvantage is closely intertwined with demographic and socioeconomic inequalities. Rather than operating independently of individual circumstances, local digital conditions may influence the extent to which residents can access and benefit from digitally mediated health resources. This interpretation is consistent with socioecological perspectives emphasizing that health behaviors are shaped by the interaction between individual resources and broader environmental conditions (Zhang and Huo, 2026; Bansal and Choudhary, 2024).

The cross-level interaction provides suggestive evidence that the association between individual digital literacy and exercise participation varies according to the broader digital environment. The positive association became weaker as area-level digital exclusion increased. However, this moderation effect was modest: the interaction OR of 0.96 indicates only a small attenuation on the multiplicative odds scale, and adding the interaction term did not improve model fit according to AIC or BIC. The interaction should therefore not be interpreted as evidence of a large contextual effect. Moreover, in logistic models, an interaction OR does not correspond to a constant absolute change in exercise participation because its substantive magnitude depends on the baseline probability of the outcome and the joint levels of the interacting variables. The predicted-probability estimates similarly suggested a somewhat flatter association between digital literacy and exercise participation under higher digital exclusion, but the absolute differences were modest. Thus, the interaction is statistically detectable and theoretically consistent with a multilevel perspective on digital inequality, while its incremental explanatory and practical contribution appears limited.

The heterogeneity analyses suggested that age was the most consistent source of variation. In particular, the negative interaction between individual digital literacy and area-level digital exclusion was stronger among adults aged 60 years and older, suggesting that older adults may be more sensitive to contextual digital constraints when translating digital capacity into exercise participation. In contrast, differences across residence, education, and household economic-resource groups were less consistently supported by formal between-group tests (Bai and Yang, 2025). These subgroup findings should therefore be interpreted cautiously and regarded as exploratory rather than definitive evidence of effect modification, particularly given the multiple subgroup comparisons.

These findings have implications for healthy-aging policy, although they should be interpreted in light of the modest effect sizes observed. Improving individual digital capacity may help some middle-aged and older adults make greater use of digitally mediated health and exercise resources, but individual-level interventions alone may be insufficient where broader digital environments remain disadvantaged. Policies could therefore combine digital-skills support with efforts to improve access to digital infrastructure, connectivity, and inclusive digital public services. However, given the observational design and the relatively small interaction effect, these policy implications should be viewed as suggestive rather than as evidence that such interventions would necessarily increase exercise participation.

Several limitations should be noted. First, although longitudinal data were used, the analysis was observational and cannot establish causality or exclude reverse causation. Second, digital literacy was measured using a CHARLS-based composite proxy rather than a validated scale. While it captures digital access, use, and functional engagement, it does not directly assess competencies such as information evaluation, problem-solving, privacy awareness, or digital confidence, and may partly reflect socioeconomic resources and technological access. The non-monotonic bivariate pattern also warrants cautious interpretation. Third, city-level digital exclusion may not capture inequalities within communities or neighborhoods. Fourth, residual confounding is possible because health, psychological, and social factors were not fully measured. Poor health, chronic disease, mobility or functional limitations may reduce both exercise participation and digital engagement, while motivation, self-efficacy, social support, loneliness, and attitudes toward technology may also influence the associations. Finally, exercise participation was self-reported and may not fully capture intensity, duration, quality, or long-term adherence.

Future research should employ validated multidimensional measures of digital literacy, more granular measures of local digital environments, and research designs better suited to causal inference, including fixed-effects approaches, natural experiments, policy evaluations, and intervention studies. Future studies should also directly investigate the behavioral and social mechanisms through which digital resources may be associated with exercise participation and examine whether these relationships differ across stages of later life and socioeconomic contexts.

## Conclusion

This study shows that individual digital literacy is positively associated with exercise participation among middle-aged and older adults in China, and that this positive association is weakened in areas with higher levels of digital exclusion. The findings indicate that digital inequality operates through both personal and contextual pathways, shaping not only access to digital resources but also the extent to which such resources can be translated into health-promoting behavior. Policies aimed at promoting healthy aging should therefore address both older adults’ digital literacy and the broader digital environments in which they live. More broadly, the study underscores the need to understand digital exclusion as a multilevel source of health inequality in rapidly aging and digitalizing societies.

## Data Availability

The original contributions presented in this study are included in this article/Supplementary material, further inquiries can be directed to the corresponding author.

## Supplementary material

Supplementary Data 1 Data supporting the findings of this study.
